# Steering the Volume of Tissue Activated With a Directional Deep Brain Stimulation Lead in the Globus Pallidus Pars Interna: A Modeling Study With Heterogeneous Tissue Properties

**DOI:** 10.3389/fncom.2020.561180

**Published:** 2020-09-25

**Authors:** Simeng Zhang, Michele Tagliati, Nader Pouratian, Binith Cheeran, Erika Ross, Erlick Pereira

**Affiliations:** ^1^Neuromodulation Division, Abbott, Plano, TX, United States; ^2^Cedars Sinai Medical Center, Los Angeles, CA, United States; ^3^Department of Neurosurgery, University of California, Los Angeles, Los Angeles, CA, United States; ^4^Research Institute of Molecular and Clinical Sciences, St. George's University of London, London, United Kingdom

**Keywords:** VTA, DBS, GPi, heterogeneous, Parkinson's disease, directional, segmented, current steering

## Abstract

**Objective:** To study the effect of directional deep brain stimulation (DBS) electrode configuration and vertical electrode spacing on the volume of tissue activated (VTA) in the globus pallidus, pars interna (GPi).

**Background:** Directional DBS leads may allow clinicians to precisely direct current fields to different functional networks within traditionally targeted brain areas. Modeling the shape and size of the VTA for various monopolar or bipolar configurations can inform clinical programming strategies for GPi DBS. However, many computational models of VTA are limited by assuming tissue homogeneity.

**Methods:** We generated a multimodal image-based detailed anatomical (MIDA) computational model with a directional DBS lead (1.5 mm or 0.5 mm vertical electrode spacing) placed with segmented contact 2 at the ventral posterolateral “sensorimotor” region of the GPi. The effect of tissue heterogeneity was examined by replacing the MIDA tissues with a homogeneous tissue of conductance 0.3 S/m. DBS pulses (amplitude: 1 mA, pulse width: 60 μs, frequency: 130 Hz) were used to produce VTAs. The following DBS contact configurations were tested: single-segment monopole (2B-/Case+), two-segment monopole (2A-/2B-/Case+ and 2B-/3B-/Case+), ring monopole (2A-/2B-/2C-/Case+), one-cathode three-anode bipole (2B-/3A+/3B+/3C+), three-cathode three-anode bipole (2A-/2B-/2C-/3A+/3B+/3C+). Additionally, certain vertical configurations were repeated with 2 mA current amplitude.

**Results:** Using a heterogeneous tissue model affected both the size and shape of the VTA in GPi. Electrodes with both 0.5 mm and 1.5 mm vertical spacing (1 mA) modeling showed that the single segment monopolar VTA was entirely contained within the GPi when the active electrode is placed at the posterolateral “sensorimotor” GPi. Two segments in a same ring and ring settings, however, produced VTAs outside of the GPi border that spread into adjacent white matter pathways, e.g., optic tract and internal capsule. Both stacked monopolar settings and vertical bipolar settings allowed activation of structures dorsal to the GPi in addition to the GPi. Modeling of the stacked monopolar settings with the DBS lead with 0.5 mm vertical electrode spacing further restricted VTAs within the GPi, but the VTA volumes were smaller compared to the equivalent settings of 1.5 mm spacing.

## Introduction

Deep brain stimulation (DBS) is an established therapy for treatment of advanced movement disorders including Parkinson's disease, tremor, and dystonia. In Parkinson's disease, in addition to the subthalamic nucleus (STN), the posterolateral “sensorimotor” region of the globus pallidus pars interna (GPi) is also targeted due to its larger size and demonstrated efficacy to improve tremor, bradykinesia, rigidity, and drug-induced dyskinesia (Bejjani et al., 1998; Williams et al., 2014; Mirza et al., 2017; Wong et al., 2019). While several studies have reported similar motor benefits when comparing GPi to STN DBS, others have reported less improvement in rigidity and bradykinesia with GPi DBS compared to STN DBS (Krack et al., 1998; Houeto et al., 2000; Okun et al., 2009; Volkmann et al., 2009). In dystonia, GPi is the DBS target of choice with demonstrated long-term efficacy and cost-benefit (Volkmann et al., 2012).

More recently, studies showing significant improvement in bradykinesia with GPi DBS examined the location of the DBS lead within the pallidum and noted that superior outcomes were associated with active contacts located in the dorsal portion of the GPi near the medial medullary lamina (Bejjani et al., 1998; Krack et al., 1998; Yelnik et al., 2000). In addition, stimulation of the external segment of the globus pallidus (GPe) has also been demonstrated to improve bradykinesia and rigidity symptoms (Vitek et al., 2004; Johnson and McIntyre, 2008; Johnson et al., 2012). Therefore, stimulating at the level of medial medullary lamina between GPi and GPe is an emerging concept in GPi programming. The exact mechanisms of GPi DBS-induced symptom relief are still under active investigation. It is likely that, similar to STN DBS where stimulation appears to activate axons leaving and adjacent to the STN (Hashimoto et al., 2003; Xu et al., 2008), a similar mechanism exists for GPi DBS (Johnson et al., 2012; Zhang et al., 2012; Muralidharan et al., 2017). Thus it is likely that stimulation of this region near the medial medullary lamina activates not only GPi motor efferents, but also axons passing through or adjacent to GPi (Parent et al., 1995; Sato et al., 2000).

Computational modeling of the volume of tissue activated (VTA) in DBS is a widely accepted technique that facilitates visualization of the affected or activated tissue areas surrounding the DBS electrode. Though it is a simplified method that does not differentiate between the activation of different neural components (i.e., cell body vs. fiber), or account for the different cell types and orientations, the VTA is generally considered to represent an “averaged” response that can be correlated with programming settings and clinical results (Dembek et al., 2017; Johnson et al., 2019; Reich et al., 2019).

Traditional VTA studies have focused on monopolar settings with ring electrodes, where a sphere-shaped activation profile is generated (Butson and McIntyre, 2008). The segmented DBS lead, which has multiple electrode segments around the lead circumference, was recently approved by the FDA for targeting STN, GPi, and the ventral intermediate nucleus of the thalamus (VIM). Recent modeling studies have now extended the VTA calculation to segmented DBS leads. Activation of a single electrode segment of these leads resulted in a shift in laterality of the VTA, sometimes known as directional DBS (Buhlmann et al., 2011; Zhang et al., 2019). However, there have been very few studies where bipolar settings have been used to model the VTA (Buhlmann et al., 2011; Duffley et al., 2019), and among those that have, homogeneous tissue models were used. Additionally, to date there has been a lack of computational modeling studies that incorporate both heterogeneous tissue properties and bipolar settings in this space.

Incorporating tissue heterogeneity and anisotropy plays an important role in shaping the VTA (Butson et al., 2007; Gunalan et al., 2017, 2018; Howell and McIntyre, 2017; Ineichen et al., 2018). When incorporating tissue heterogeneity, the electric field changes from spherical to irregular shapes that are stimulation target-dependent (Ineichen et al., 2018). Additionally, according to vector analyses of electric field isolevels, compared to other DBS targets, the GPi has the greatest angles of deviation as a result of tissue heterogeneity and anisotropy (Aström et al., 2012). Taken together these findings provide compelling evidence to suggest that the actual VTA is not spherical, and more physiologically and anatomically accurate models are necessary to more precisely model tissue activation.

By leveraging our previous work calculating VTAs in the STN (Zhang et al., 2019), we hereby report a computational model for VTAs in the globus pallidus (GP) using directional leads. Here, we demonstrate the utility and potential advantages of using two vertical electrode spacing options (0.5 mm and 1.5 mm) with various monopolar and bipolar settings, and their effects on the size and shape of the resultant VTA in a heterogeneous tissue model. This study provides a simple framework to guide the selection of lead segments/contacts and programming parameters to sculpt the VTA in order to target two example regions of the pallidum: the posterolateral “sensorimotor” GPi, or GPi and GPe at the level of medial medullary lamina.

## Methods

### Finite Element Models

A finite element model (FEM) of the human head was implemented in Sim4Life v4.0 with the multimodal image-based detailed anatomical (MIDA) model following the methodology described in our previous publication (Zhang et al., 2019). Since in the original MIDA model, the GP was not segmented into the internal and external segments, we performed a manual segmentation by overlaying the MNI atlas onto the MIDA GP (performed with FSL), and then segmenting the region into 3 sub-regions: GPi, GPe, and medial medullary lamina (area between GPe and GPi). A segmented DBS lead (Infinity, Abbott) with 0.5 mm or 1.5 mm vertical inter-electrode spacing was placed in the globus pallidus (23 degrees toward anterior direction in sagittal plane and 11 degrees toward lateral direction in coronal plane with segmented electrode 2A facing anterior), with segmented contact 2 in the ventral posterolateral portion of the left GPi. The surface of contact 2 was ~2.25 mm away from the lateral boarder and 2.3 mm from the posterior boarder of the GPi (Supplementary Figure 1). A 0.5 mm thick encapsulation layer was added around the lead (Anderson et al., 2019). The electrical conductivity of the brain tissues, platinum-iridium contacts on the DBS lead, and polyurethane insulation on the lead were determined from the IT'IS database 3.1.1 (DATABASE ≫ IT'IS Foundation)^1^. To demonstrate the effect of tissue heterogeneity on the FEM, as an example, a homogeneous tissue model was also used to calculate VTAs for configuration 4 (see next section) by replacing the entire internal structures of the head with homogeneous tissue with a 0.3 S/m conductance (Geddes and Baker, 1967).

Electrical potentials were calculated using various contact configurations by setting the boundaries of the active contacts to a voltage-controlled condition (Dirichlet boundary condition). The return electrode (anode) of the monopolar stimulation was represented using the boundaries of the epidermis layer in the MIDA head model. A bounding box of size 175.2 x 227.5 x 251.5 mm that encompassed all other model structures was modeled with zero normal current density (Neumann boundary condition). To determine the equivalent current delivered, the total current flux was calculated over the boundary of the cathode(s). Given the input voltage and the current flux on the cathode(s), an impedance of the electrode-tissue interface (ETI) was calculated and the equivalent current delivered was computed.

A rectilinear, volumetric mesh grid was generated from the model geometries with 0.04 mm maximum edge size for the electrodes, 0.1 mm max edge size for structures near the electrode (in a region of interest of 27 x 20 x 23 mm^3^), and 5 mm maximum step size elsewhere (over 98 million elements total). Convergence was set to a relative value of 1e-8 and an absolute value of 1e-10. Finally, an electromagnetic ohmic quasi-static solver was used to solve the following equation at the mesh nodes at the given current amplitude and frequency:

(1) $$\begin{array}{r} \nabla \cdot \bar{\epsilon }\nabla \varphi =0 \end{array}$$

where $\bar{\epsilon }$ is the complex electric permittivity, φ is the electric potential, and:

(2) $$\begin{array}{r} \bar{\epsilon }=\epsilon_{R}\epsilon_{0}+\frac{\sigma }{j\omega } \end{array}$$

where ϵ_*R*_ is the relative permittivity, ϵ_0_ is the relative permittivity of perfect vacuum, and σ is the electrical conductivity.

Multi-compartment axons that were 20 mm in length and 5.7 μm diameter were distributed on axonal planes that were perpendicular to the lead and 0.5 mm apart from one another. Within each plane, the axons were arranged parallel to one another with 0.25 mm spacing and rotated 5 times by 30 degrees per rotation. The electrical potentials from the FEM were interpolated along each neuron and delivered as extracellular stimulation to determine which axons were activated for a given contact configuration and stimulation set. All neuronal activations were computed in Sim4Life.

### DBS Parameters and Configurations

**Figure 1:**
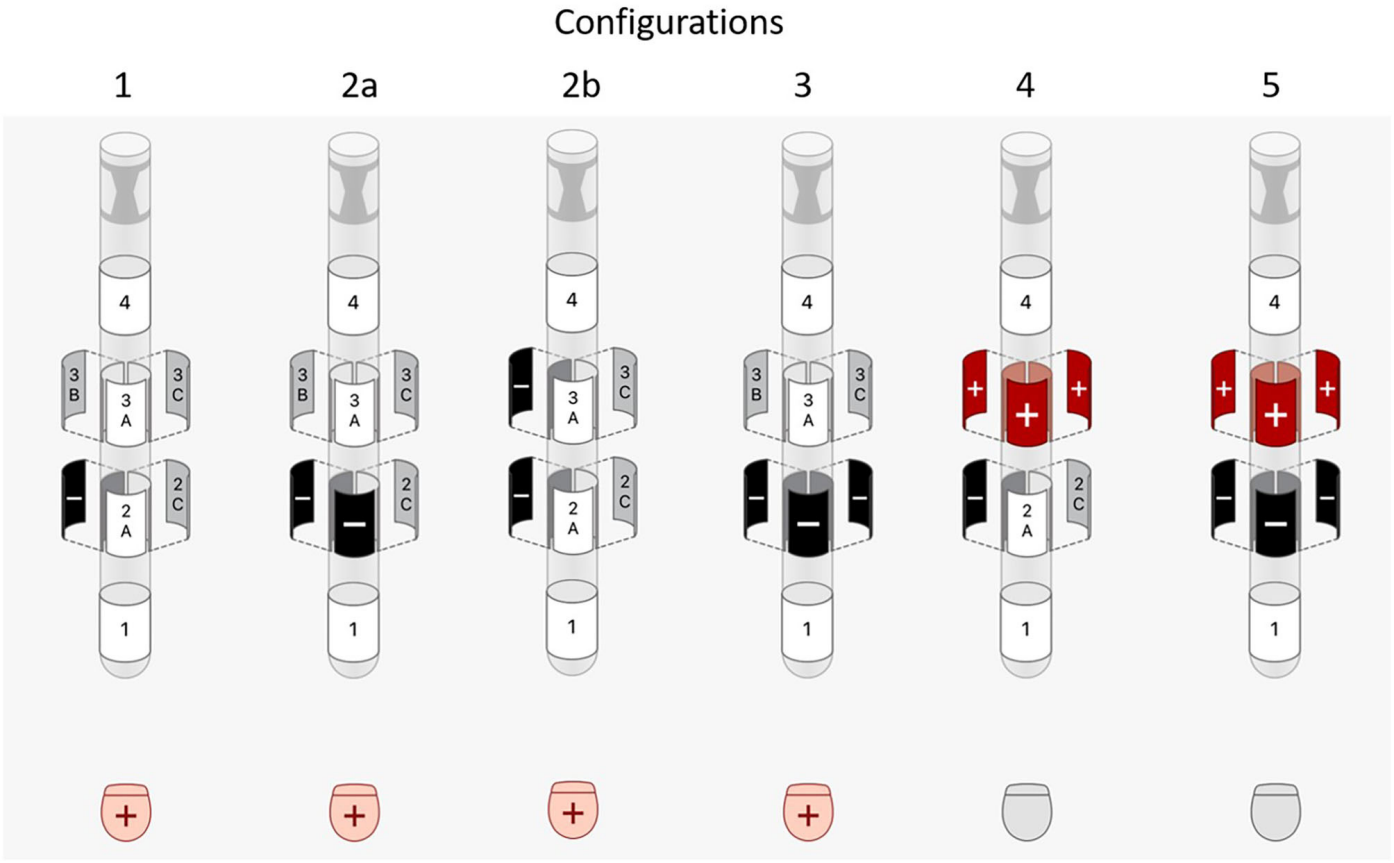
Illustrations of the DBS programming configurations tested.

DBS pulses of 1 mA with 60 μs pulse width (biphasic with passive discharge) and 130 Hz were used when modeling VTAs. Since electrode 2A was facing anterior, electrode 2B was determined to be the most optimal electrode for activation of posterolateral GPi. Therefore, the following common DBS contact **configurations** were tested (Figure 1):

1. single-segment monopole (2B-/Case+)
2. two-segment monopole
  - row (2A-/2B-/Case+)
  - vertically stacked (2B-/3B-/Case+)
3. ring monopole (2A-/2B-/2C-/Case+)
4. one-cathode-ring-anode bipole (2B-/3A+/3B+/3C+)
5. ring-cathode-ring-anode bipole (2A-/2B-/2C-/3A+/3B+/3C+).

Configuration 2b, 4, and 5, which contain vertically activated segments, were repeated for a directional DBS lead with 0.5 mm vertical electrode spacing. In addition, all configurations were repeated using an amplitude of 2 mA.

All configurations that contained two or more segments were simulated as if the electrodes were connected via hardware parallel connections. This is sometimes referred to as “co-activation” and is a common method of activation when the DBS system only has a single current source.

### Volume of Tissue Activated Generation

The volumes of tissue activated (VTA) were calculated in Matlab R2017b according to previously described methods by bounding the action potential initiation (API) sites in space to form a 3D volume (Zhang et al., 2019). The 3D volume was then sub-divided into four volumes: (1) inside GPi, (2) between GPi and GPe, (3) inside GPe, and (4) outside GP, by partitioning the VTA into voxels in each region.

## Results

### Effect of Tissue Heterogeneity

**Figure 2:**
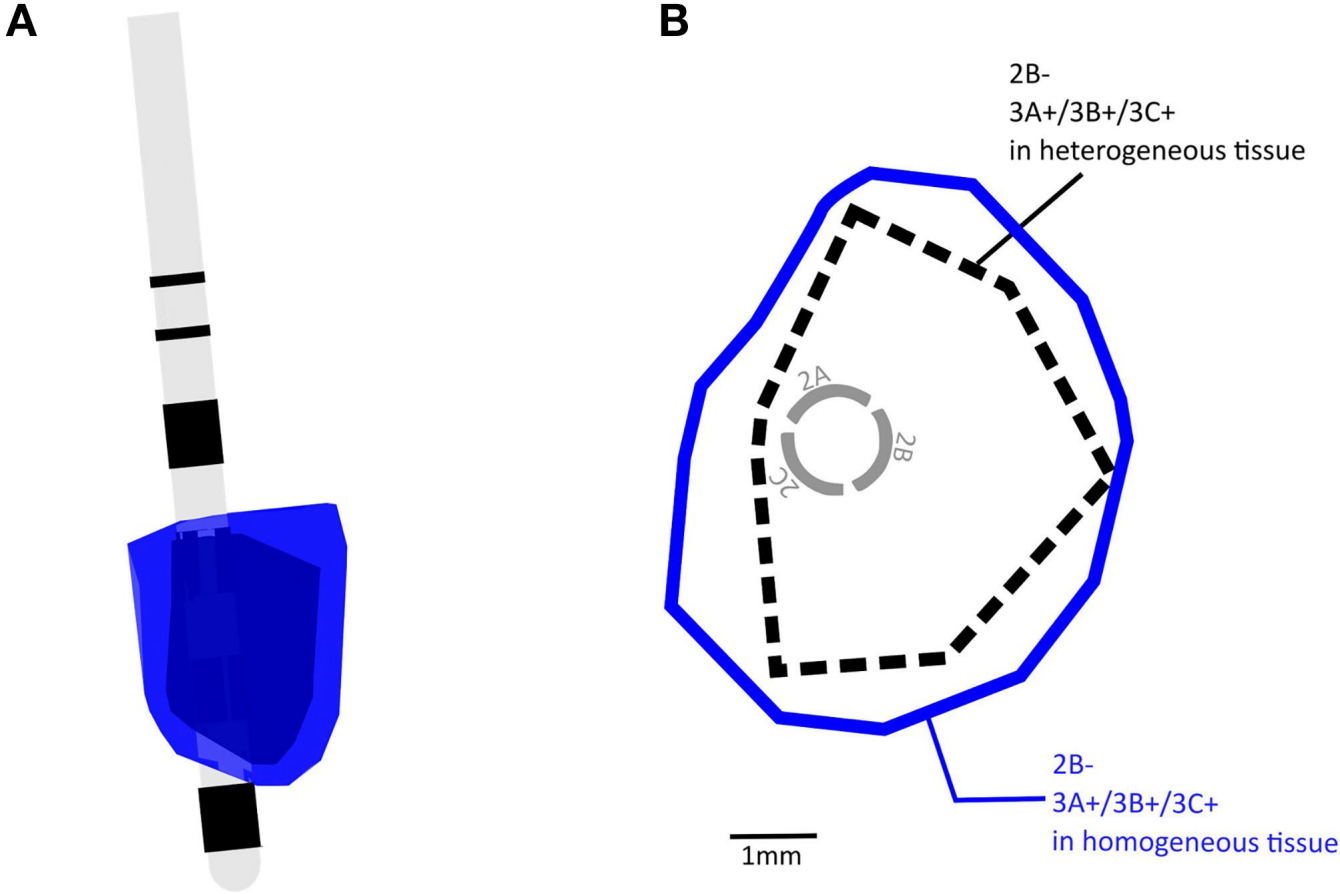
**(A)** Examples of overlapping VTA volumes calculated with homogeneous tissue and heterogeneous tissue conductance, blue = homogeneous tissue, black = heterogeneous tissue. **(B)** A transverse view showing boundaries of VTAs shown in **(A)**.

Consistent with previous findings, replacing the heterogeneous MIDA model with a homogeneous model for configuration 4 resulted in VTAs that were 42.9% larger in size and more regular in shape at the cross sections (Butson et al., 2007; Gunalan et al., 2017, 2018; Howell and McIntyre, 2017; Ineichen et al., 2018). The VTA for configuration 4 in a homogeneous and heterogeneous model is shown in Figure 2 and the transverse cross-sectional outline in 2B. We used the heterogeneous tissue model to calculate all remaining VTAs in this study.

### VTA Volume

**Figure 3:**
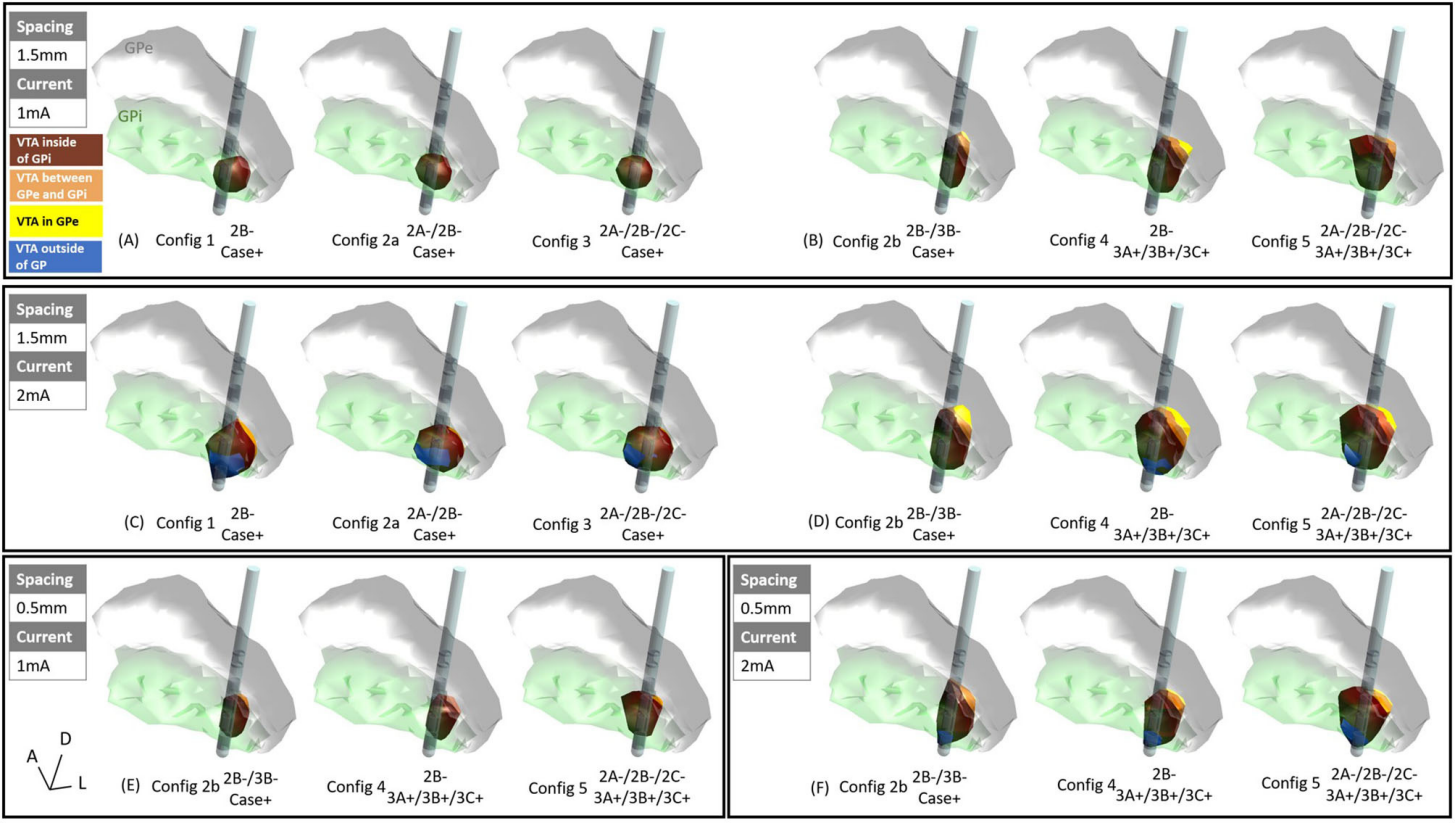
VTAs for various configurations: **(A)** 1.5 mm vertical spacing with 1 mA current, settings 1, 2a, and 3 (see methods), **(B)** 1.5 mm vertical pacing with 1 mA current, settings 2b, 4, and 5, **(C)** 1.5 mm vertical spacing with 2 mA current, settings 1, 2a, and 3, **(D)** 1.5 mm vertical pacing with 2 mA current, settings 2b, 4, and 5, **(E)** 0.5 mm vertical spacing with 1 mA current, settings 2b, 4, and 5, **(F)** 0.5 mm vertical spacing with 2 mA current, settings 2b, 4, and 5, marron = VTA inside of GPi, orange = VTA between GPe and GPi (medial medullary lamina), yellow = VTA inside of GPe, blue = VTA outside of GP.

When the active electrode was placed near the ventral posterolateral “sensorimotor” GPi, and at low current settings such as 1 mA current, the VTAs produced by configurations 1, 2a, and 3 (Figure 1) were entirely within the GPi (Figure 3A). However, as the current increases from 1 to 2 mA, VTAs enlarged and exceeded the GPi boundary from the ventral side (and sometimes medial side) into undesired side effect regions such as the optic tract or the internal capsule (Figure 3C, blue). Consistent with previous findings, the VTAs with the single-segment monopole (configuration 1) generated the most axially asymmetric and largest VTA at the cathodic contact (Zhang et al., 2019).

For the vertically stacked two-segment monopole configuration (configuration 2b), at 1 mA current amplitude, the VTA elongated dorsally along the lead and activates more structures dorsal to the GPi such as the medial medullary lamina (Figure 3B, orange) and GPe (Figure 3B, yellow). Note that because the vertical electrodes segments are stacked facing the same direction, the directionality of the VTA was the same for stacked two-segment configuration 2b as the single segment configuration 1. A similar activation profile was also observed for vertical bipolar settings (configurations 4 and 5), where VTAs extended to dorsal structures to the GPi (for 1.5 mm spacing: Figure 3B, and for 0.5 mm spacing: Figure 3E). At 2 mA, the VTA volume expanded, also exceeding the ventral border involving regions associated with the development of side effects (blue regions in Figures 3D,F). However, the VTAs of regions associated with side effects were on average 86.6% smaller in configurations 2b, 4, and 5 (Figures 3D,F) than those in configurations 1, 2a, and 3 (Figure 3C).

**Figure 4:**
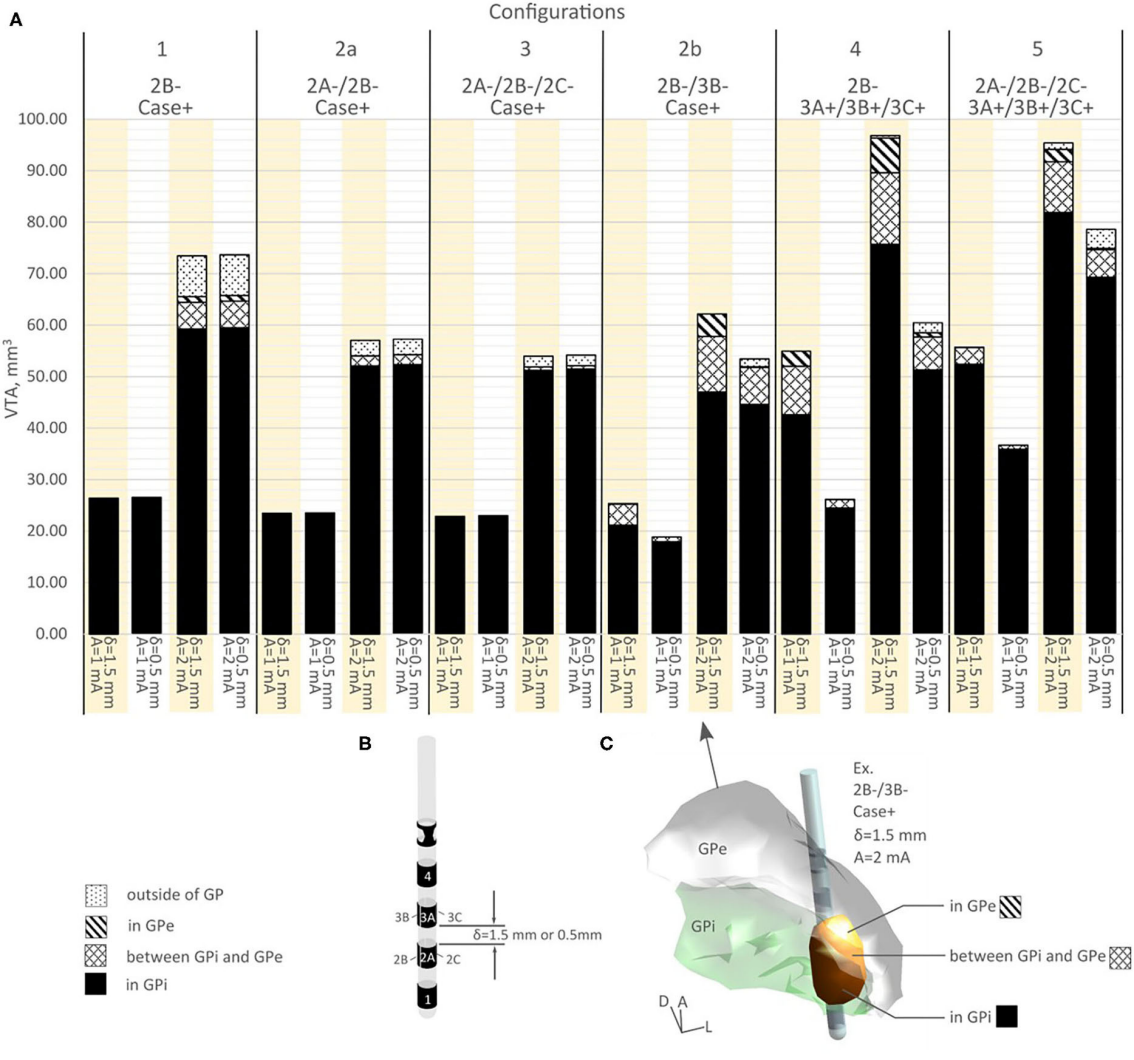
**(A)** VTA volume for various configurations in different structures. **(B)** A segmented DBS lead showing various vertical spacing. **(C)** A visual example of VTA volume distribution.

A detailed volume break-down of the VTAs in each of the sub-regions is summarized in Figure 4 (Supplementary Table 1). Because configurations 1, 2a, and 3 did not engage any vertical electrode combinations, these three configurations produced the same VTA distribution between the 1.5 mm DBS electrode and the 0.5 mm DBS electrode (Figure 4A, Supplementary Table 1). In configurations 2b, 4, and 5, compared to the 1.5 mm lead, the 0.5 mm DBS lead produced smaller VTAs with the same current amplitude. The resulting VTA was much more concentrated in GPi. At 1 mA, configurations 2b, 4, and 5 with 0.5 mm vertical electrode spacing produced a VTA that was on average 95.39% within the GPi, while 1.5 mm vertical spacing produced a VTA that was on average 85.01% in GPi. Similarly, at 2 mA current amplitude, configurations 2b, 4, and 5 with 0.5 mm vertical electrode spacing produced a VTA that was on average 85.45% in the GPi, while a 1.5 mm vertical spacing produced VTA that was on average 79.86% in GPi.

In the bipolar configurations, switching from configuration 4 to 5, the 1.5 mm vertical spacing DBS lead produced similar VTAs (1.31% increase in volume for 1 mA current, −1.48% decrease in volume for 2 mA current, see Figure 4A and Supplementary Table 1). However, the composition of volumes activated changed switching from configuration 4 to 5, as the % volume in GPi increased from 77.47 to 94.15% for 1 mA, and from 78.17 to 85.83% for 2 mA (Figure 4A, Supplementary Table 1). For the 0.5 mm DBS lead, both the volume and the % volume within GPi increased when switching from configurations 4 to 5. The % volume in GPi increased from 93.53 to 97.71% for 1 mA current amplitude, and from 84.79 to 88.16% for 2 mA. The VTA volumes increased an average of 35.31% switching from configuration 4 to 5.

## Discussion

This study explored the effect of tissue heterogeneity and various electrode montages on the VTA to help inform directional programming of the GPi. In particular, the effects of vertical electrode spacing in combination with commonly used monopolar and bipolar settings on VTAs in both GPi and structures dorsal to the GPi were examined.

### Strategies of Programming GPi DBS Based on VTAs

To maximize therapeutic response when targeting the posterolateral GPi, the goal is to maximize the VTA within the GPi while minimizing VTA extension to off target regions that are associated with side effects, such as the internal capsule or optic tract. In this study, we found that if a DBS electrode is placed well within the sensorimotor territory of the GPi, using a single segment of the DBS electrode (configuration 1), two active segments (configuration 2a), or ring mode (configuration 3) can all produce VTAs that are entirely within the GPi at low current amplitudes (1 mA in this study). Among the three configurations, single segment activation (configuration 1) produced the largest VTA. We also observed that if the electrode was placed closer to regions that could cause side effects we could restrict the VTA by use a single segment (configuration 1) to steer the VTA toward the desired region of interest since single segment activation produced a VTA with maximum axial asymmetry (Zhang et al., 2019).

Another way to maximize GPi activation without extension into neighboring structures such as GPe, optic tract or internal capsule, while offering more flexible selections of configurations may be to use a DBS lead with 0.5 mm vertical spacing. At low current amplitudes, in addition to the 3 above mentioned configurations, users can also use vertical stacked monopolar settings (configuration 2b) and vertical bipolar settings (configurations 4, 5).

Vertical configurations (configurations 2b, 4, 5) are generally helpful to stretch the VTA and involve a greater dorsal-to-ventral extent of the target that could include the dorsal pallidum structures such as the medial medullary lamina and/or ventral portions of GPe. Additionally, one can opt to use a 1.5 mm vertically spaced DBS lead to further elongate and enlarge the VTA to reach the dorsal pallidum structures. Correspondingly, if the user wants to increase volume of VTA inside of GPi compared to dorsal structures, one can switch to the 0.5 mm vertical spacing DBS electrode, or switch from using a monopole as cathode in a bipolar setting (configuration 4) to using a ring as cathode in a bipolar setting (configuration 5), as doing so will increase the VTA volume at the level of the cathode located inside of GPi. Table 1 summarized these findings.

**Table 1:** Summary of strategies of programming GPi DBS based on VTAs.

**Desired activation target**	**Additional criteria**	**Recommended vertical spacing**	**Recommended configurations**
Posterolateral “sensorimotor” GPi only	-	0.5 mm or 1.5 mm	1, 2a, 3
	-	0.5 mm	2b, 4, 5
GPi, medial medullary lamina and/or GPe	less GPi activation	1.5 mm	2b, 4
	more GPi activation	0.5 mm	2b, 5

Note that the electrode with 1.5 mm vertical spacing consistently resulted in larger VTA volume than the one with 0.5 mm vertical spacing for the parameters tested. This can potentially mean that using a DBS lead with 1.5 mm vertical spacing can activate a given volume of GPi and dorsal pallidal structures with less current amplitude compared to the lead with 0.5 mm vertical spacing.

### Limitations of Bipolar VTA

The VTA method used in this paper was a direct bounding method, which bounded the API of axons of various orientations. This is different from the “center node remapping” method, where the APIs were remapped to the center node of the activated axons. The two methods did not differ significantly for cathodal stimulation, as the API tend to be the node that is closest to the cathode (Anderson et al., 2019), but because of the virtual cathode effect, the axons near the anode tend to initiate APIs that are further toward the distal ends, rather than the node that is closest to the anode (Slopsema et al., 2018; Anderson et al., 2019). The direct bounding VTA method used here therefore resulted in VTAs that were larger at the anode than previously reported. There was evidence that previously reported method likely underestimated the activations near the anode (Slopsema et al., 2018; Anderson et al., 2019; Duffley et al., 2019), therefore, the method used here that incorporates multiple orientations orthogonal to the lead offered a more directly interpretable VTA activation profile, especially for a larger target such as the globus pallidus, and especially with a lead with larger vertical spacing (1.5 mm). However, a bigger VTA produced by bipolar stimulations does not directly translate to better therapy or equate with lower side effect threshold—the shape of the VTA matters more in terms of overlapping with therapy regions and side effect pathway activations.

Axonal fiber orientation also matters for bipolar stim (Slopsema et al., 2018; Anderson et al., 2019). The axonal grid used in this study contained axons that were perpendicular to the DBS lead, but in the GP, there are fibers that run parallel to the lead, and future simlations should certainly include parallel fibers in the model. This resulted in an under-estimation of the axonal activation. However, this effect could be compensated by the choice of using 4.7 um axon fibers, where most of the fibers in GP have smaller fiber diameter. Overall, The effects of using the direct bounding VTA method, not including parallel fibers, and using 4.7 um axons should counter-balance each other and produce a reasonable VTA estimation.

### Other Limitations and Future Directions

In this study, we examined commonly used electrode activation configurations for DBS in the GP. We did not examine many other possible activation configurations such as multipolar stimulation or using one segment as the anode, as those configurations are not commonly practiced in the clinic. Future studies might include those configurations for a complete assessment of the VTAs in GPi DBS. Additionally, this current study only used 1 mA and 2 mA as stimulation current amplitudes as examples to offer guidance on the VTA size and inform programming. The clinician will still need to increase and titrate the current levels to obtain the best therapeutic effects, and the end results will be highly dependent on the location and orientation of the electrode. Additionally, the parameters simulated here alone were not enough for visually aided programming—since VTA is not linearly correlated to the input current amplitude, further subdivision of current is needed to produce the finer VTAs that can be used for visual programming.

One other limitation of this study is that only tissue heterogeneity was reflected with different conductance values. Tissue anisotropy was not incorporated, which might play an even bigger role than tissue conductance alone (Aström et al., 2012). In future studies we plan to introduce tissue anisotropy into the MIDA model. Additionally, we plan to implement a patient-specific version of the MIDA model that is extracted based on the patient's imaging data. The electrode location and orientation will be detected by postoperative CTs and co-registered to the MRIs to produce a more accurate representation of the VTA in individual patients.

## Conclusion

We demonstrated for the first time using a heterogenous tissue conductance computational model that if the traditional posterolateral “sensorimotor” GPi is the target, depending on lead placement, using one or more electrode segments of the same ring with the optimal current level can achieve a VTA the incorporates a significant region of the sensorimotor GPi without current spread into adjacent areas. Using a single segment also produced a VTA with the largest volume. Using stacked vertical two-cathode settings produced a VTA that expanded the VTA in the dorsal-to-ventral direction and could be used to include regions dorsal to the GPi. Alternatively, vertical bipolar settings can also effectively enlarge the VTA at the anode without activating areas ventral to the cathode. We also showed that with these settings and a lead well placed within the sensorimotor territory of the GPi, using a DBS electrode with 0.5 mm vertical electrode spacing would be beneficial for restricting a greater percentage of the VTA to the GPi, while a 1.5 mm vertical electrode spacing could be used to expand the VTA volume and extend the VTA to more dorsal regions.

## Data Availability Statement

The datasets presented in this article are not readily available because only computational models were used. Requests to access the datasets should be directed to david.zhang@abbott.com.

## Author Contributions

SZ, BC, and EP: conception and design of study. SZ: acquisition of data and drafting the manuscript. All authors: analysis and/or interpretation of data, revising the manuscript critically for important intellectual content, and approval of the version of the manuscript to be published.

## Conflict of Interest

SZ, BC, and ER are employees and receive salary from Abbott. MT, NP, and EP consult for Abbott and receives compensation for their time. NP also serves as a consultant for Medtronic, Boston Scientific, Second Sight Medical Products and receives grand support from Second Sight Medical Products and BrainLab. MT also serves as a consultant for Medtronic, Boston Scientific and Revance. The authors declare that this study received funding from Abbott. The funder was not involved in the study design, collection, analysis, interpretation of data, the writing of this article or the decision to submit it for publication.

## Footnotes

^1^DATABASE. IT'IS Foundation. Available online at: https://itis.swiss/virtual-population/tissue-properties/database/ (accessed August 31, 2018).
