# Supplementary material for: Steering the Volume of Tissue Activated With a Directional Deep Brain Stimulation Lead in the Globus Pallidus Pars Interna: A Modeling Study With Heterogeneous Tissue Properties
Source: Front Comput Neurosci. 2020 Sep 25;14:561180. doi: 10.3389/fncom.2020.561180 (PMC7546409; doi:10.3389/fncom.2020.561180)
Supplement: Supplementary file 1 [file Table_1.DOCX]

Supplementary Material

**Supplementary Table 1. VTA volumes calculated for all configurations**

| Configurations | Vertical spacing | Current | Volume, mm^3^ | | | | |
| --- | --- | --- | --- | --- | --- | --- | --- |
|  |  |  | Total | In Gpi | Between GPi and GPe | In GPe | Outside of GP |
| 2B-/Case+ | 1.5mm | 1mA | 26.40 | 26.40 | 0.00 | 0.00 | 0.00 |
| 2A-/2B-/Case+ |  |  | 23.44 | 23.44 | 0.00 | 0.00 | 0.00 |
| 2A-/2B-/2C-/Case+ |  |  | 22.86 | 22.86 | 0.00 | 0.00 | 0.00 |
| 2B-/3B-/Case+ |  |  | 25.35 | 21.15 | 4.06 | 0.14 | 0.00 |
| 2B-/3A+/3B+/3C+ |  |  | 54.94 | 42.56 | 9.48 | 2.90 | 0.00 |
| 2A-/2B-/2C-/3A+/3B+/3C+ |  |  | 55.66 | 52.40 | 3.24 | 0.02 | 0.00 |
| 2B-/Case+ | 1.5mm | 2mA | 73.64 | 59.44 | 5.17 | 1.12 | 7.91 |
| 2A-/2B-/Case+ |  |  | 57.20 | 52.20 | 1.94 | 0.05 | 3.01 |
| 2A-/2B-/2C-/Case+ |  |  | 54.10 | 51.39 | 0.62 | 0.00 | 2.09 |
| 2B-/3B-/Case+ |  |  | 62.32 | 47.11 | 10.85 | 4.36 | 0.00 |
| 2B-/3A+/3B+/3C+ |  |  | 97.10 | 75.91 | 13.93 | 6.79 | 0.48 |
| 2A-/2B-/2C-/3A+/3B+/3C+ |  |  | 95.66 | 82.10 | 9.88 | 2.43 | 1.25 |
| 2B-/Case+ | 0.5mm | 1mA | 26.40 | 26.40 | 0.00 | 0.00 | 0.00 |
| 2A-/2B-/Case+ |  |  | 23.44 | 23.44 | 0.00 | 0.00 | 0.00 |
| 2A-/2B-/2C-/Case+ |  |  | 22.86 | 22.86 | 0.00 | 0.00 | 0.00 |
| 2B-/3B-/Case+ |  |  | 18.71 | 17.76 | 0.95 | 0.00 | 0.00 |
| 2B-/3A+/3B+/3C+ |  |  | 26.01 | 24.33 | 1.69 | 0.00 | 0.00 |
| 2A-/2B-/2C-/3A+/3B+/3C+ |  |  | 36.58 | 35.74 | 0.84 | 0.00 | 0.00 |
| 2B-/Case+ | 0.5mm | 2mA | 73.64 | 59.44 | 5.17 | 1.12 | 7.91 |
| 2A-/2B-/Case+ |  |  | 57.20 | 52.20 | 1.94 | 0.05 | 3.01 |
| 2A-/2B-/2C-/Case+ |  |  | 54.10 | 51.39 | 0.62 | 0.00 | 2.09 |
| 2B-/3B-/Case+ |  |  | 53.35 | 44.50 | 7.18 | 0.16 | 1.52 |
| 2B-/3A+/3B+/3C+ |  |  | 60.40 | 51.22 | 6.41 | 0.82 | 1.95 |
| 2A-/2B-/2C-/3A+/3B+/3C+ |  |  | 78.58 | 69.28 | 5.34 | 0.31 | 3.66 |
