# Supplementary material for: Steering the Volume of Tissue Activated With a Directional Deep Brain Stimulation Lead in the Globus Pallidus Pars Interna: A Modeling Study With Heterogeneous Tissue Properties
Source: Front Comput Neurosci. 2020 Sep 25;14:561180. doi: 10.3389/fncom.2020.561180 (PMC7546409; doi:10.3389/fncom.2020.561180)
Supplement: Supplementary file 2 [file Data_Sheet_1.PDF]

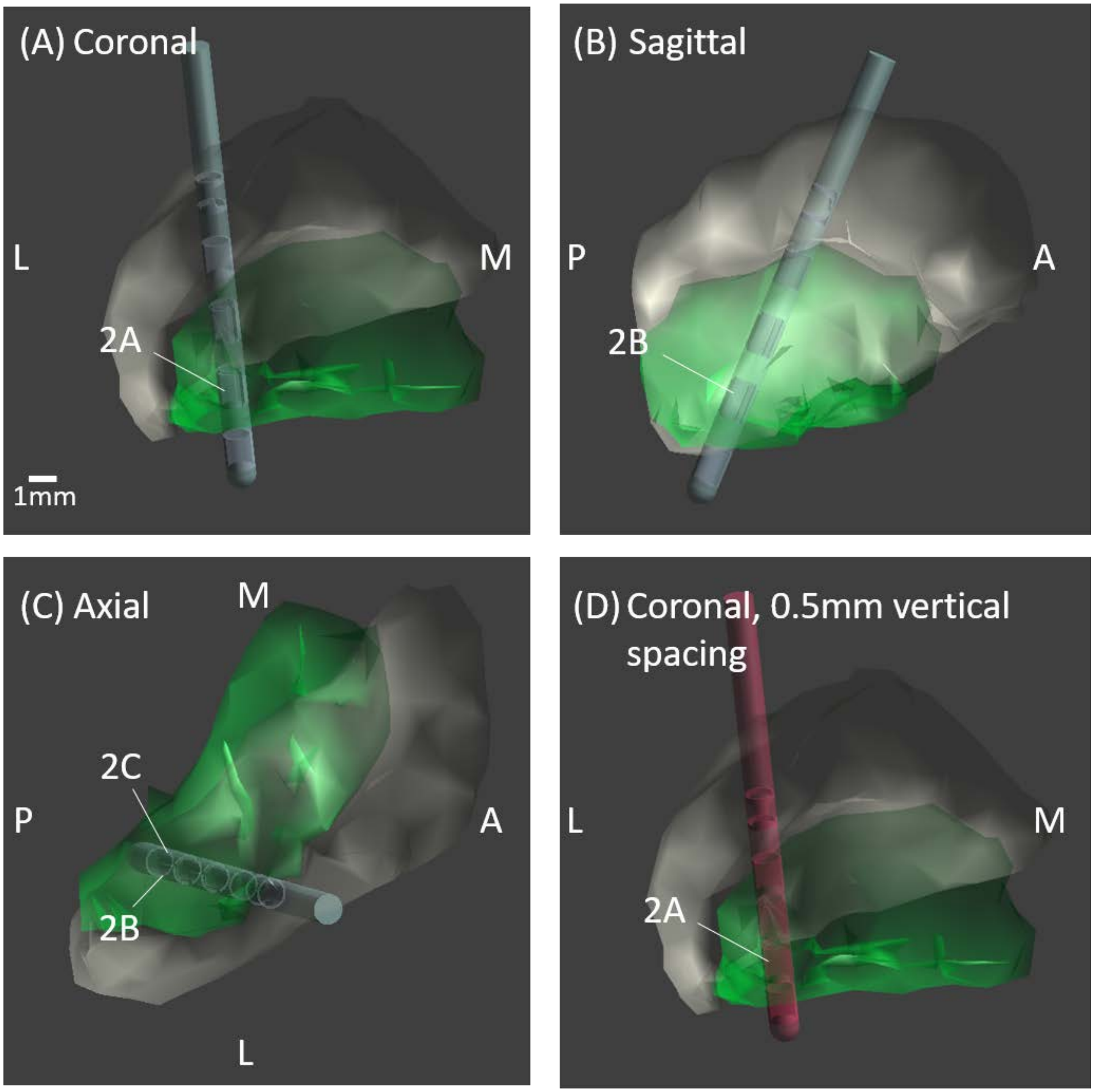

Supplementary Figure 1. (A) Coronal, (B) Sagittal and (C) Axial views of the electrode location within the globus pallidus, showing a segmented lead with 1.5mm vertical spacing. (D) Coronal view showing a segmented lead with 0.5mm vertical spacing.
